# Supplementary material for: Loss of sea ice alters light spectra for aquatic photosynthesis
Source: Nat Commun. 2025 Apr 30;16:4059. doi: 10.1038/s41467-025-59386-x (PMC12043827; doi:10.1038/s41467-025-59386-x)
Supplement: Supplementary file 1 — Supplementary Information [file 41467_2025_59386_MOESM1_ESM.pdf]

Supplementary Information

Loss of sea ice alters light spectra for aquatic photosynthesis

Monika Soja-Woźniak[1], Tadzio Holtrop[1,2], Sander Woutersen[3], Hendrik Jan van der Woerd[2], Lars Chresten Lund-Hansen[4] and Jef Huisman[1*]*

[1]Department of Freshwater and Marine Ecology, University of Amsterdam, Amsterdam, the Netherlands
[2]Department of Water & Climate Risk, Institute for Environmental Studies, VU University Amsterdam, Amsterdam, the Netherlands
[3]Van't Hoff Institute for Molecular Sciences, University of Amsterdam, Amsterdam, The Netherlands
[4]Department of Biology, Arctic Research Centre, Aquatic Biology, Aarhus University, Aarhus, Denmark
*Corresponding authors: Monika Soja-Woźniak (monika@soja-wozniak.com) and Jef Huisman (j.huisman@uva.nl)

Table of Contents

Table S1: Parameter values and functions used in the radiative transfer model to generate the model predictions in Figure 2.

Table S2: Parameter values and functions used in the radiative transfer model to generate the model predictions in Figures 3-5 and Supplementary Figures S3-5.

Table S3: Contribution of scattering to total light attenuation in sea ice and in liquid water.

Figure S1: Spectra of incident solar irradiance measured at the Central Arctic Ocean, the Baltic Sea, and McMurdo Sound (Antarctica).

Figure S2: Spectrum of incident solar irradiance used as input for the model to calculate the scalar irradiance spectra shown in Figures 3-5 and Supplementary Figures S3-5.

Figure S3: Sensitivity of the model predictions to the albedo of sea ice.

Figure S4: Sensitivity of the model predictions to the scattering coefficient of sea ice.

Figure S5: Sensitivity of the model predictions to snow cover.

Figure S6: Schematic diagram illustrating the model structure for sea ice.

Supplementary References.

Table S1: Parameter values and functions used in the radiative transfer model to generate the model predictions in Figure 2. Parameters were measured by the indicated references.

User-defined IOP model			
	**Components**	**Absorption spectrum**	**Scattering spectrum**
Central Arctic Ocean[2,35]	Sea ice[#]<br>*albedo[35] = 55%*<br>*thickness[35] = 1.53 m*	Warren & Brandt (2008)[71]<br>[plotted in Figure 1b]	SSL: $b_{ice}(\lambda) = 400$ m^{-1} for 0 < z ≤ 0.05 m<br>DL: $b_{ice}(\lambda) = 75$ m^{-1} for 0.05 < z ≤ 0.40 m<br>INT: $b_{ice}(\lambda) = 24$ m^{-1} for 0.40 < z ≤ 1.53 m
	Seawater	for λ >550 nm: Pope & Fry (1997)[23]<br>for λ <550 nm: Mason et al. (2016)[25]<br>[plotted in Figure 1b]	Morel (1974)[22]<br>[plotted in Figure 1a]
	Chlorophyll-*a*<br>*in ice[35,†]: 0.01 mg m^{-2}*<br>*in water[35]: 0.12 mg m^{-3}*	Prieur & Sathyendranath (1981)[79]	Gordon & Morel (1983)[80]
	CDOM	ice: $a_{CDOM}(\lambda) = 0.1\, e^{-0.007(\lambda-440)}$ m^{-1}<br>water[35]: $a_{CDOM}(\lambda) = 0.27\, e^{-0.007(\lambda-440)}$ m^{-1}	0
	NAP[‡]	Matsuoka et al. (2011)[76]<br>$a_{NAP}(\lambda) = 0.01\, e^{-0.007(\lambda-440)}$ m^{-1}	NAP[74,76] = 0.32 g m^{-3},<br>using the average of mass-specific scattering spectra for calcareous sand, yellow clay, red clay and brown earth[33]
Baltic Sea[34]	Sea ice[#]<br>*albedo[34,*] = 21%*<br>*thickness[34] = 0.19 m*	Warren & Brandt (2008)[71]<br>[plotted in Figure 1b]	SSL: $b_{ice}(\lambda) = 2$ m^{-1} for 0 < z ≤ 0.01 m<br>DL: $b_{ice}(\lambda) = 1$ m^{-1} for 0.01 < z ≤ 0.05 m<br>INT: $b_{ice}(\lambda) = 0.5$ m^{-1} for 0.05 < z ≤ 0.19 m
	Seawater	for λ >550 nm: Pope & Fry (1997)[23]<br>for λ <550 nm: Mason et al. (2016)[25]<br>[plotted in Figure 1b]	Morel (1974)[22]<br>[plotted in Figure 1a]
	Chlorophyll-*a*<br>*in ice[34†]: 1.0 mg m^{-2}*<br>*in water[34]: 0.4 mg m^{-3}*	Prieur & Sathyendranath (1981)[79]	Gordon & Morel (1983)[80]
	CDOM	ice[34]: $a_{CDOM}(\lambda) = 0.39\, e^{-0.014(\lambda-440)}$ m^{-1}<br>water[34]: $a_{CDOM}(\lambda) = 1.27\, e^{-0.014(\lambda-440)}$ m^{-1}	0
	NAP	ice[34]: $a_{NAP}(\lambda) = 0.05\, e^{-0.007(\lambda-440)}$ m^{-1}<br>water[34]: $a_{NAP}(\lambda) = 0.006\, e^{-0.007(\lambda-440)}$ m^{-1}	ice[34]: NAP = 1.65 g m^{-3}<br>water[34]: NAP = 0.18 g m^{-3}<br>using the average of the mass-specific scattering spectra for calcareous sand, yellow clay, red clay and brown earth[33]
Mc Murdo Sound Antarctica[2]	Sea ice[#]<br>*albedo[$] = 65%*<br>*thickness[$] = 1.98 m*	Warren & Brandt (2008)[71]<br>[plotted in Figure 1b]	SSL: $b_{ice}(\lambda) = 400$ m^{-1} for 0 < z ≤ 0.05 m<br>DL: $b_{ice}(\lambda) = 80$ m^{-1} for 0.05 < z ≤ 0.50 m<br>INT: $b_{ice}(\lambda) = 40$ m^{-1} for 0.50 < z ≤ 1.98 m
	Seawater	for λ >550 nm: Pope & Fry (1997)[23]<br>for λ <550 nm: Mason et al. (2016)[25]<br>[plotted in Figure 1b]	Morel (1974)[22]<br>[plotted in Figure 1a]
	Chlorophyll-*a*<br>*in ice[2,†]: 15.2 mg m^{-2}*<br>*in water[$]: 1.1 mg m^{-3}*	Prieur & Sathyendranath (1981)[79]	Gordon & Morel (1983)[80]
	CDOM[#]	$a_{CDOM}(\lambda) = 0.06\, e^{-0.014(\lambda-440)}$ m^{-1}	0
	NAP[‡]	Matsuoka et al. (2011)[76]<br>$a_{NAP}(\lambda) = 0.01\, e^{-0.007(\lambda-440)}$ m^{-1}	NAP[74,76] = 0.32 g m^{-3},<br>using the average of the mass-specific scattering spectra for calcareous sand, yellow clay, red clay and brown earth[33]

[#] *Scattering coefficients of sea ice ($b_{ice}(\lambda)$) at all three locations and absorption by CDOM ($a_{CDOM}(440)$) at McMurdo Sound, Antarctica, were estimated by least-squares fits of the modelled spectra to the measured spectra.*

[†] *Chlorophyll-a concentrations in ice were expressed per unit surface area (as in refs 2,34); the model used ice thickness to convert these values to chlorophyll-a concentrations per unit volume.*

[‡] *The NAP concentrations at the Central Arctic Ocean and McMurdo Sound, Antarctica, were not reported in Lund-Hansen et al.[2,34]; instead, we used $a_{NAP}(440)$=0.01 m^{-1} measured in the Arctic Ocean by Matsuoka et al.[76].*

[*] *The albedo of the Baltic Sea was based on reflectance spectra of bare ice measured by Kari et al.[34], where we used the lowest values of the range; this was equivalent to an albedo of 21% when averaged over the spectrum.*

[$] *Measured by L.C. Lund-Hansen, this study.*

Table S2: Parameter values and functions used in the radiative transfer model to generate the model predictions in Figures 3-5 and Supplementary Figures S3-5. Parameters were measured by the indicated references, parameters without a reference were estimated by this study. SSL = surface scattering layer, DL = drained layer, INT = interior layer.

User-defined IOP model		
**Components**	**Absorption spectrum**	**Scattering spectrum**
Sea ice<br>*albedo = 50%*	Warren & Brandt (2008) [71]<br>[plotted in Figure 1b]	SSL: $b_{ice}(\lambda) = 400\ \mathrm{m}^{-1}$<br>DL: $b_{ice}(\lambda) = 50\ \mathrm{m}^{-1}$<br>INT: $b_{ice}(\lambda) = 15\ \mathrm{m}^{-1}$<br>Light et al. (2015)[31]
Seawater<br>*albedo[83] = 6%*	for λ >550 nm: Pope & Fry (1997)[23]<br>for λ <550 nm: Mason et al. (2016)[25]<br>[plotted in Figure 1b]	Morel (1974)[22]<br>[plotted in Figure 1a]
Chlorophyll-*a*	0	0
CDOM	Full range from very low[75] to very high[42,43] CDOM concentrations:<br>$a_{CDOM}(\lambda) = a_{CDOM}(440)\ e^{-0.014(\lambda-440)}$ m^{-1}<br>with $a_{CDOM}(440) \in (0.0025, 5)$	0
NAP	Default value for clear ocean water[74]:<br>$a_{NAP}(\lambda) = 0.001\ e^{-0.007(\lambda-440)}$ m^{-1}	NAP[74,76] = 0.032 g m^{-3},<br>using the average of the mass-specific scattering spectra for calcareous sand, yellow clay, red clay and brown earth[33]
**Semi-empirical sky model**		
Solar zenith angle =65°	Cloud cover=0%	Day of Year=228
**Bottom Boundary Condition: infinitely deep water column**		

Table S3: Contribution of scattering to total light attenuation in sea ice and in open water. The contribution of scattering is calculated as the single-scattering albedo* at five wavelengths (400, 450, 550, 700 and 850 nm), for the three different marine ecosystems displayed in Figure 4. SSL = surface scattering layer, DL = drained layer, INT = interior layer.

Single-scattering albedo*							
		SEA ICE			OPEN WATER		
		Oligotrophic ocean	Mesotrophic ocean	Coastal water	Oligotrophic ocean	Mesotrophic ocean	Coastal water
$a_{CDOM}(440)$[#]		0.01 m^{-1}	0.1 m^{-1}	1 m^{-1}	0.01 m^{-1}	0.1 m^{-1}	1 m^{-1}
400 nm	SSL	0.9999	0.9996	0.9958			
	DL	0.9996	0.9966	0.9673	0.2855	0.0455	0.0048
	INT	0.9987	0.9887	0.8987			
450 nm	SSL	0.9999	0.9998	0.9979			
	DL	0.9998	0.9982	0.9834	0.2312	0.0542	0.0063
	INT	0.9992	0.9942	0.9468			
550 nm	SSL	0.9999	0.9998	0.9993			
	DL	0.9988	0.9985	0.9948	0.0428	0.0331	0.0101
	INT	0.9962	0.9949	0.9828			
700 nm	SSL	0.9986	0.9986	0.9986			
	DL	0.9893	0.9892	0.9888	0.0019	0.0019	0.0019
	INT	0.9651	0.9649	0.9635			
850 nm	SSL	0.9930	0.9930	0.9930			
	DL	0.9467	0.9467	0.9467	0.0002	0.0002	0.0002
	INT	0.8420	0.8420	0.8419			

* *The single-scattering albedo (ω_0) represents the proportion of the light attenuation that is attributed to scattering. Its value is determined by the equation $\omega_0 = b(\lambda)/[a(\lambda) + b(\lambda)]$, where $a(\lambda)$ is the total absorption coefficient and $b(\lambda)$ is the total scattering coefficient as function of wavelength λ. The single-scattering albedo is dimensionless, with a value of 1 if all attenuation is due to scattering, and a value of 0 if all attenuation is due to absorption. Hence, the calculated values show that light scattering prevails over light absorption in sea ice, while absorption prevails over scattering in open waters.*

[#] *$a_{CDOM}(440)$ is the absorption coefficient of coloured dissolved organic matter (CDOM) at 440 nm.*

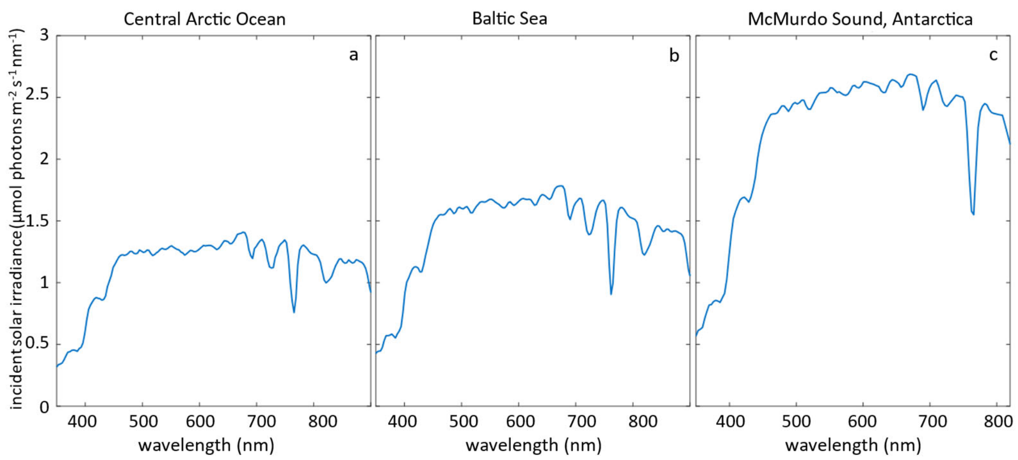

Figure S1: Spectra of incident solar irradiance measured at the Central Arctic Ocean, the Baltic Sea, and McMurdo Sound (Antarctica). (**a**) Central Arctic Ocean in August 2012, (**b**) the Baltic Sea (Bothnian Bay) on 6th March 2016, and (**c**) McMurdo Sound, Antarctica, in early November 2014. These three incident spectra were used as input to the model to predict the planar irradiance spectra below sea ice shown in Figure 2. The spectra in (**a**) and (**c**) are from Lund-Hansen et al.[2] and in (**b**) from Kari et al.[34]

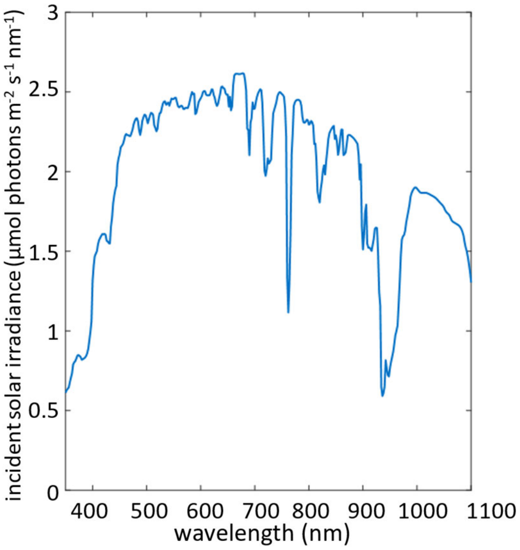

Figure S2: Spectrum of incident solar irradiance used as input for the model to calculate the scalar irradiance spectra shown in Figures 3-5 and Supplementary Figures S3-5. The spectrum was measured at the Arctic Ocean by Grenfell & Perovich[84] (see their Figure 5), on the 16th of August 2005, when the sky was clear and the solar disk was clearly visible. The solar elevation angle was 25.4°.

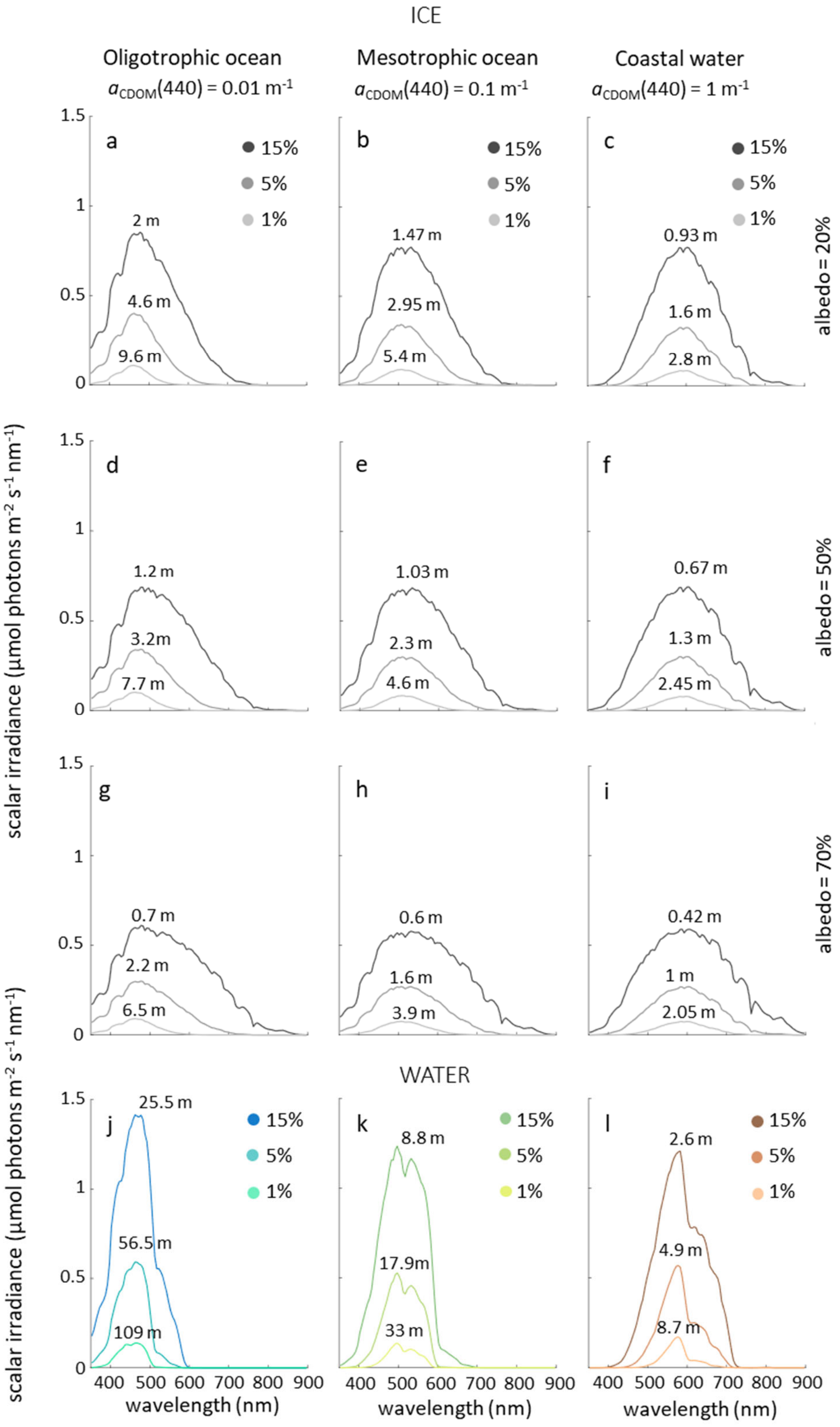

Figure S3: Sensitivity of the model predictions to the albedo of sea ice. (**a-i**) Scalar irradiance spectra directly under the ice (gray lines), calculated for (**a-c**) low albedo (20%), (**d-f**) intermediate albedo (50%), and (**g-i**) high albedo (70%) of sea ice. (**j-l**) Scalar irradiance spectra in open water (coloured lines). The spectra are shown for three marine ecosystems with different CDOM concentrations: the oligotrophic ocean (left column), mesotrophic ocean (middle column), and eutrophic coastal waters (right column). In each panel, irradiance spectra are shown at three optical depths at 15%, 5% and 1% of the incident irradiance, as indicated by the colours of the lines. The associated physical depths (in meters) are also indicated, representing the thickness of the ice in (**a-i**) and the depth in the water column in (**j-l**). The parameter settings are summarized in Supplementary Table S2.

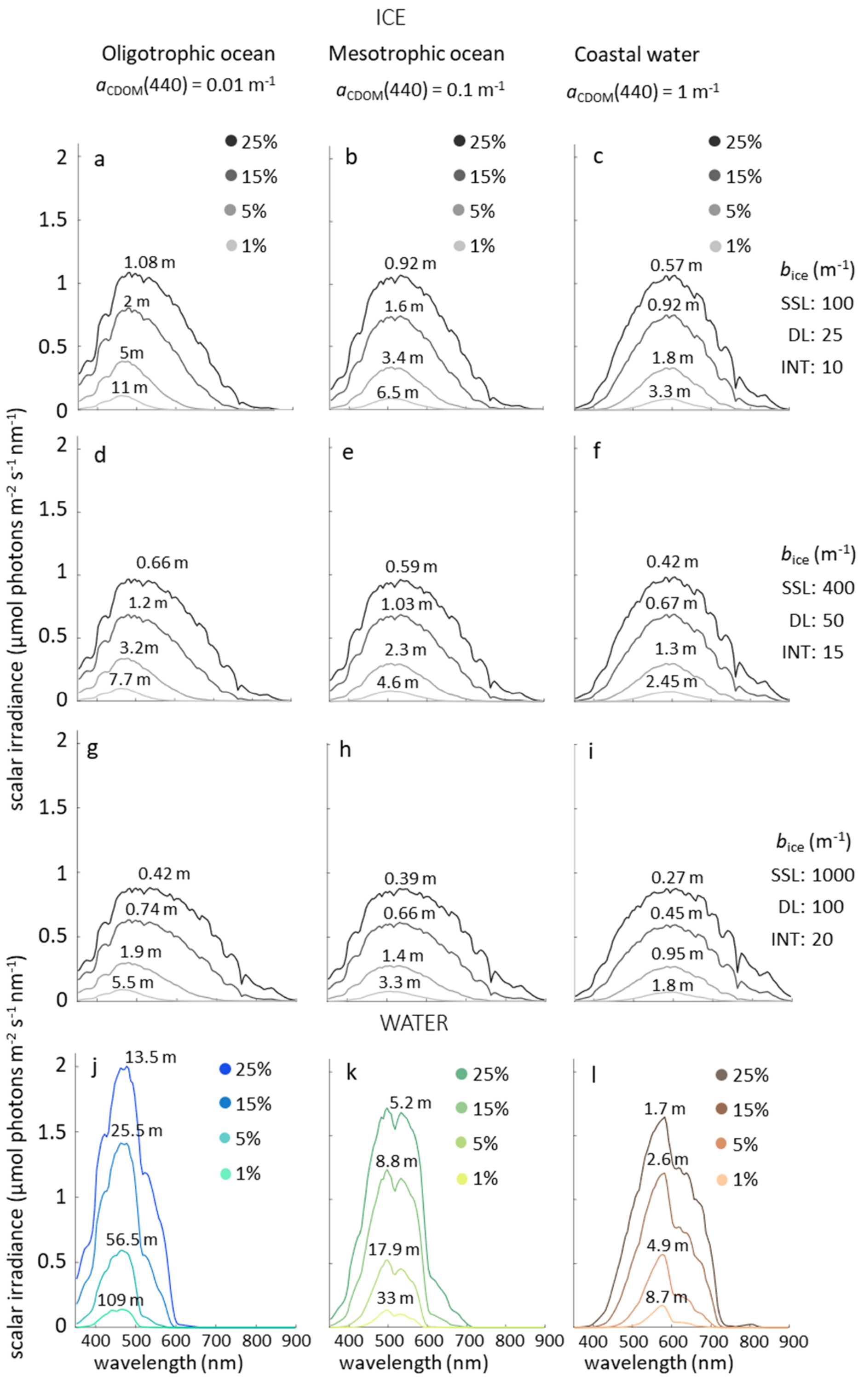

Figure S4: Sensitivity of the model predictions to the scattering coefficient of sea ice. Because scattering of sea ice varies with depth, the model assumes that ice consists of three layers: the surface scattering layer (SSL), drained layer (DL), and interior layer (INT) (see Figure S6). (**a-i**) Scalar irradiance spectra directly under the ice (gray lines), calculated for (**a-c**) low values, (**d-f**) intermediate values, and (**g-i**) high values of the scattering coefficient of sea ice (b_{ice}) in these three layers (as indicated at the right-hand side of the graphs). (**j-l**) Scalar irradiance spectra in open water (coloured lines). The spectra are shown for three marine ecosystems with different CDOM concentrations: the oligotrophic ocean (left column), mesotrophic ocean (middle column), and eutrophic coastal waters (right column). In each panel, irradiance spectra are shown at four optical depths at 25%, 15%, 5% and 1% of the incident irradiance, as indicated by the colours of the lines. The associated physical depths (in meters) are also indicated, where the physical depth represents the thickness of the ice in (**a-i**) and the depth in the water column in (**j-l**). The parameter settings are summarized in Supplementary Table S2.

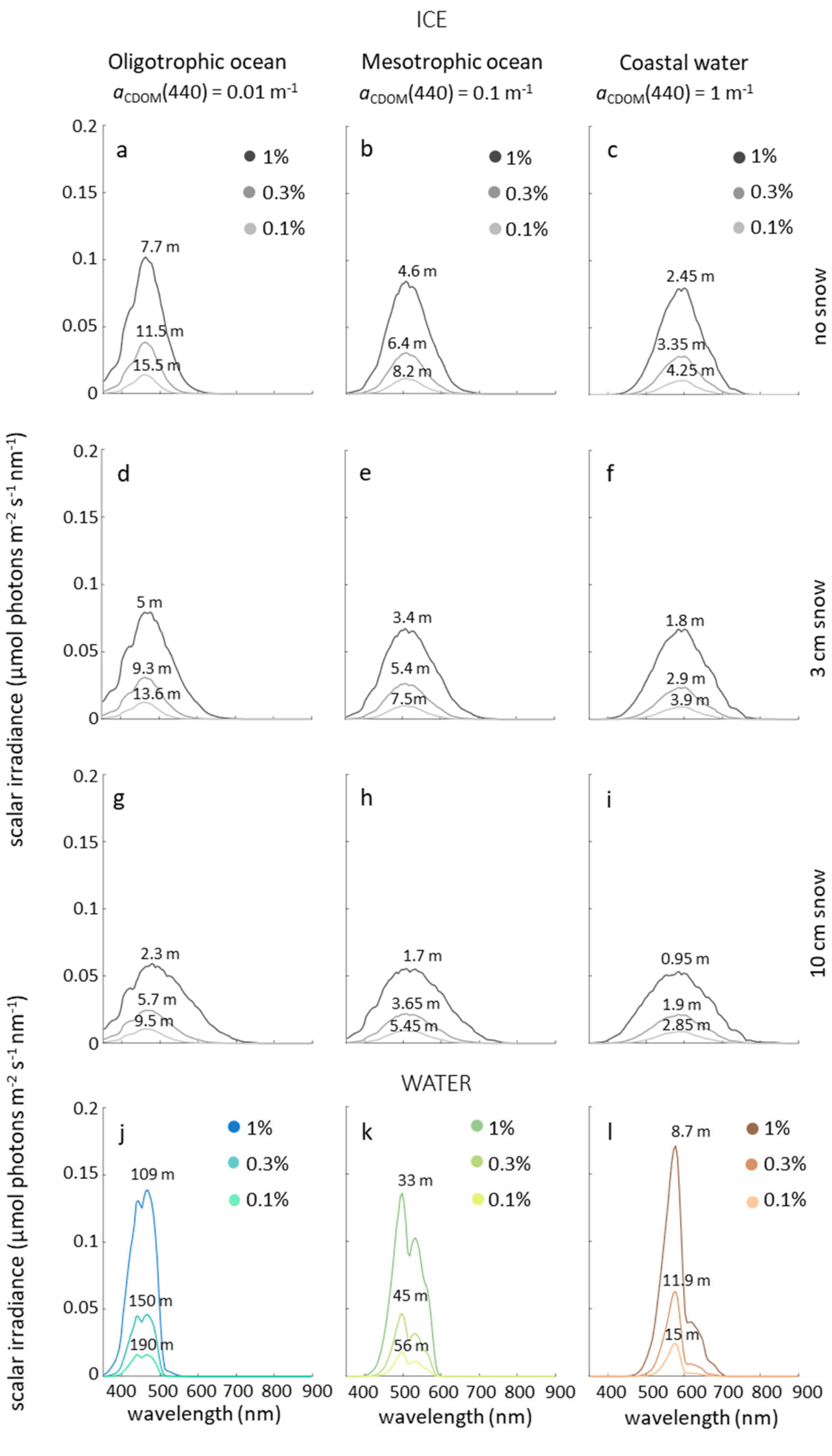

Figure S5: Sensitivity of the model predictions to snow cover. (**a-i**) Scalar irradiance spectra directly under the ice (gray lines), for (**a-c**) ice without snow cover, (**d-f**) ice with 3 cm of snow, (**g-i**) ice with 10 cm of snow. (**j-l**) Scalar irradiance spectra in open water (coloured lines). We assume that ice without snow cover consists of three layers, the surface scattering layer (SSL), drained layer (DL) and interior layer (INT), whereas snow-covered ice consists of a snow layer on top of an interior layer of ice (INT)[19]. The spectra are calculated for three marine ecosystems with different CDOM concentrations: the oligotrophic ocean (left column), mesotrophic ocean (middle column), and eutrophic coastal waters (right column). In each panel, irradiance spectra are shown at the same three optical depths at 1%, 0.3% and 0.1% of the incident irradiance, as indicated by the colours of the lines. The associated physical depths (in meters) are also indicated, where physical depth represents the thickness of ice in (**a-i**) and the depth in the water column in (**j-l**). We used the albedo and wavelength-dependent extinction coefficient of snow in Figures 3 and 5 of Perovich[37], taking into account the thickness of the snow. All other parameter settings are summarized in Supplementary Table S2.

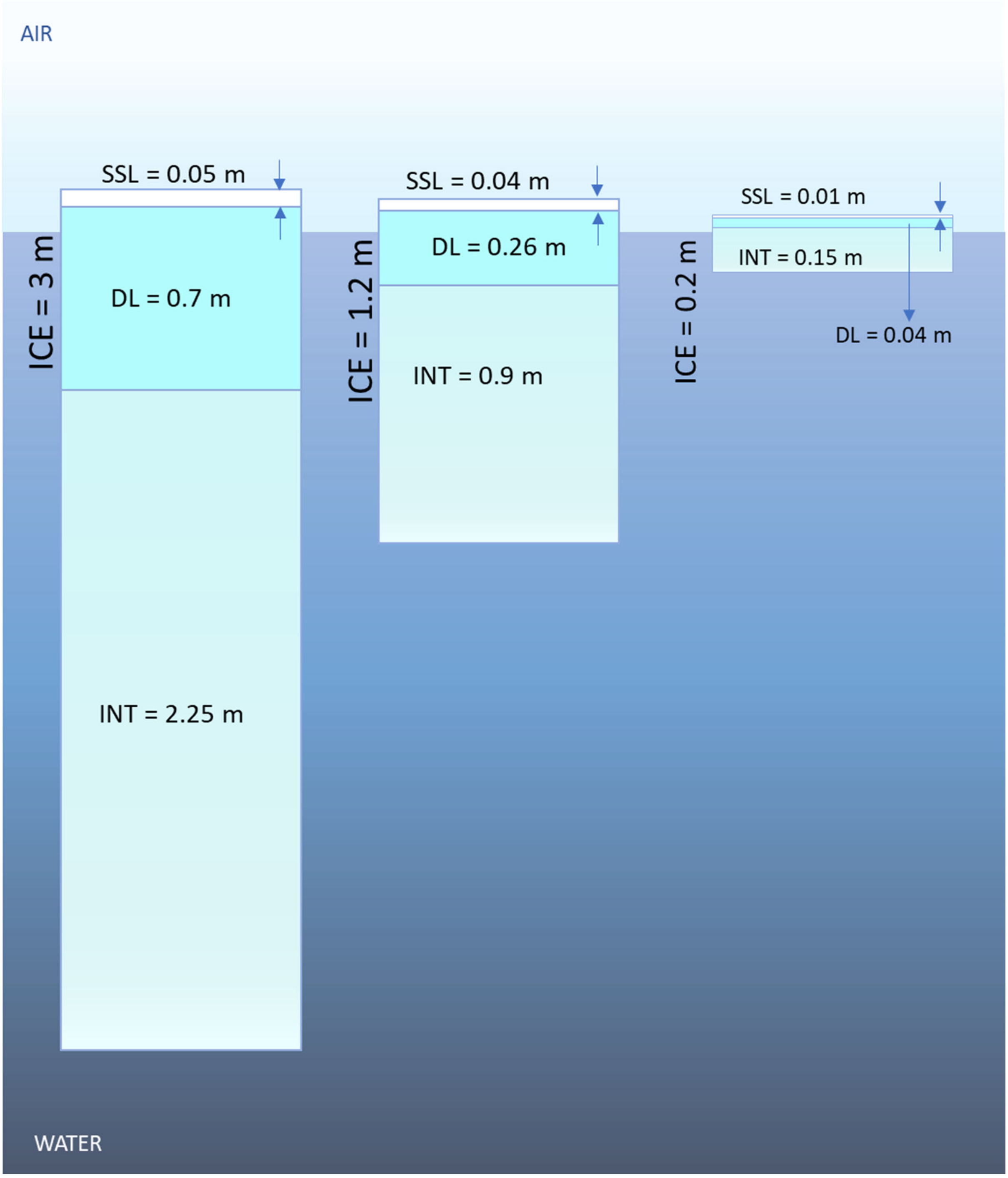

Figure S6: Schematic diagram illustrating the model structure for sea ice. Because scattering of sea ice varies with depth, the model consists of three layers[19,69]: the surface scattering layer (SSL), drained layer (DL), and interior layer (INT). The thickness of these layers depends on the total thickness of the sea ice (z_{ice}), and is calculated as follows: SSL = 0.05 m for z_{ice} > 1.5 m (left column); SSL = (1/30)z_{ice} for 1.5 > z_{ice} > 0.30 m (middle column); SSL = 0.01 m for z_{ice} < 0.30 m (right column); DL = (1/4)z_{ice} – SSL; INT = (3/4)z_{ice}.

Supplementary References

83. Payne, R. E. Albedo of the sea surface. *J. Atmos. Sci.* **29**, 959–970 (1972).

84. Grenfell, T. C. & Perovich, D. K. Incident spectral irradiance in the Arctic Basin during the summer and fall. *J. Geophys. Res. Atmos.* **113**, 2007JD009418 (2008).
